# The Screening of Celiac Serology in Pediatric Patients Diagnosed with Type 1 Diabetes Mellitus

**DOI:** 10.5152/tjg.2023.22775

**Published:** 2023-03-01

**Authors:** Didem Gülcü Taşkın, Aysun Ata

**Affiliations:** 1 Department of Pediatric Gastroenterology, Adana City Training and Research Hospital, Adana, Turkey; 2 Department of Pediatric Endocrinology, Adana City Training and Research Hospital, Adana, Turkey

**Keywords:** Celiac antibody, pediatrics, type 1 diabetes mellitus

## Abstract

**Background:**

This study aims to detect the rate of celiac antibody test positivity in pediatric patients diagnosed with type 1 diabetes mellitus (T1DM) and determine the characteristics of the patient groups diagnosed with and without celiac disease (CD).

**Methods:**

This study was conducted retrospectively in Adana City Training and Research Hospital, Pediatric Endocrinology Outpatient Clinic with the patients diagnosed with T1DM between September 17, 2017, and January 1, 2022. The patients were examined by 3 different groups. Group 1: celiac patient group, group 2: serology false positive group, and group 3: serology negative group.

**Results:**

The study included 418 patients, 228 (54.5%) males and 190 (45.5%) females. About 6% of the patients (25 patients) were in the celiac patient group, 12.6% (53 patients) in the serology false positive group, and 81.3% (340 patients) in the serology negative group. The age at diagnosis was 10 (7.2-12.9) years in the celiac patient group and 8.8 (6.2-12.00) years in the serology false positive group (*P* = .559). Among 53 patients in the serology false positive group, spontaneous normalization was observed in 66% (35 patients), and positivity was continued during the test follow-up period in 34% (18 patients). There was no significant difference in terms of sex (*P* = .101), and HbA1c values at diagnosis (*P* = .557). Tissue transglutaminase IgA titer was 20× Upper limit of normal (ULN) in the celiac patient group and 2.52× ULN in the serology false positive group.

**Conclusion:**

T1DM and CD are both autoimmune diseases concurrently seen together. CD antibody positivity may be observed at the first presentation of T1DM. While the majority of these antibodies become negative during the follow-up, we wanted to highlight the false positive antibody titers and emphasize that these patients should be followed by endocrinologists and gastroenterologists together.

**Main Points**

- Celiac disease antibody positivity may be seen as much more common at the first presentation of type 1 diabetes mellitus (T1DM), even if not accompanied by celiac disease.
- The majority of the positive antibodies become negative during the follow-up period. It is crucial to initiate the diabetic diet program of the T1DM patients and follow-up on the symptoms and the antibody normalization.
- The symptoms resolve in a significant proportion of the patients by the time under the diet program. Therefore, we recommend not doing an invasive procedure like an endoscopic examination for those patients early at the first presentation of the disease.

## Introduction

Celiac disease (CD) is an autoimmune enteropathy caused by immunological mechanisms developing after gluten consumption in genetically susceptible individuals and resulting in villous damage. While the frequency of CD differs by country, it has been reported to be approximately 1%. Similar to CD, type 1 diabetes mellitus (T1DM) is an autoimmune disease due to pancreatic damage resulting from immunological mechanisms. The frequency of CD may be up to 10-fold in T1DM patients (3%-10%) compared to the general population.^1-3^

Diagnosis is mainly made by villous atrophy shown in small intestine biopsy collected by gastroscopy in patients with clinical suspicion or serologic test positivity.^4^ However, patients diagnosed with T1DM are routinely screened for CD even if they do not have any symptoms at diagnosis. Criteria for definitive diagnosis after screening were developed in 2020 by The European Society for Paediatric Gatsroenterology Hepatology and Nutrition (ESPGHAN). According to these criteria, the diagnosis can be made in individuals with proven genetic susceptibility without biopsy if they have a 10-fold rise in tissue transglutaminase IgA (TTG IgA) titer and anti-endomysium IgA (EMA IgA) antibody positivity.^5^

High rates of false positivity have been reported in CD screening in individuals diagnosed with T1DM.^6^ False positive blood tests lead to additional work-up for the patients and place an additional burden on the healthcare system. This study aims to detect the rate of celiac antibody test positivity in pediatric patients diagnosed with T1DM and determine the characteristics of the patient groups diagnosed with and not diagnosed with CD.

## Materials and Methods

This study was designed as a single-center, retrospective study and conducted in Adana City Training and Research Hospital, Pediatric Endocrinology Clinic. A total of 418 patients who were diagnosed with T1DM and treated between January 1, 2008, and January 1, 2022, were included in the study. The antibodies studied during the CD screening of the cases were recorded by date and titer. Patients diagnosed with CD using either ESGHAN 2012^7^ or 2020^5^ guidelines were included in the “celiac patient group” (CPG) (group 1). Patients who had at least 1 positive test result for celiac antibodies after screening but without CD were included in the “serology false positive group” (SFPG) (group 2). Patients who had no positive result for any of the antibodies studied were included in the “serology negative group” (SNG) (group 3). Approval for the study was taken from Adana City Training and Research Hospital Ethics Committee. The study was designed in line with the Helsinki Declaration. An informed consent form was obtained from patients or their legal guardians.

### Statistical Analysis

Statistical analysis of the study was performed using Statistical Package for Social Sciences (SPSS) 21.0 software (IBM Corp.; Armonk, NY, USA). Categorical variables were summarized as number and percentage, and continuous variables as mean and standard deviation (median and minimum-maximum, if necessary). For the comparison of categorical variables, chi-square and Fisher’s exact tests were used. For the comparison of continuous variables, distribution normality was tested, and Mann–Whitney *U* test was used for non-normally distributed parameters. Statistical significance level, *P* < .05, was used for all tests.

## Results

Out of 418 patients included in the study, 228 (54.5%) were males and 190 (45.5%) were females. When the patients were examined by 3 different groups, 6% of the patients (25 patients) were detected to be in CPG, 12.6% (53 patients) in SFPG, and 81.3% (340 patients) in SNG. The mean duration of follow-up was 3.2 ± 2.8 years. Follow-up duration was detected to be 2.3 ± 1.8 years in CPG, 4.1 ± 2.4 years in SFPG, and 3.3 ± 2.9 years in SNG. In the patient groups included in the study, the age at diagnosis was 10 (7.2-12.9) years in CPG with the age at diagnosis in SFPG being similar, 8.8 (6.2-12.00) years (*P* = .559).

Among 53 patients in SFPG, spontaneous normalization was observed in 66% (35 patients), and positivity was continued during the test follow-up period in 34% (18 patients).

When the number of patients diagnosed with Hashimoto’s thyroiditis was examined, there were 25 patients (7.4%) in SNG, 2 patients (8%) in CPG, and 8 patients (15.1%) in SFPG (*P* = .071). While the rate of Hashimoto’s thyroiditis is higher in SFPG, the difference was not statistically significant. Patient characteristics are summarized in Table 1.

There was no significant difference in terms of sex between 3 groups (*P* = .101). All cases had similar HbA1c values at diagnosis [CPG: 13.2% (10.9-14.5), SFPG: 12.7% (10-14.4), SNG: 12.1% (10.2-13.9)] (*P* = .557).

**Figure 1.**
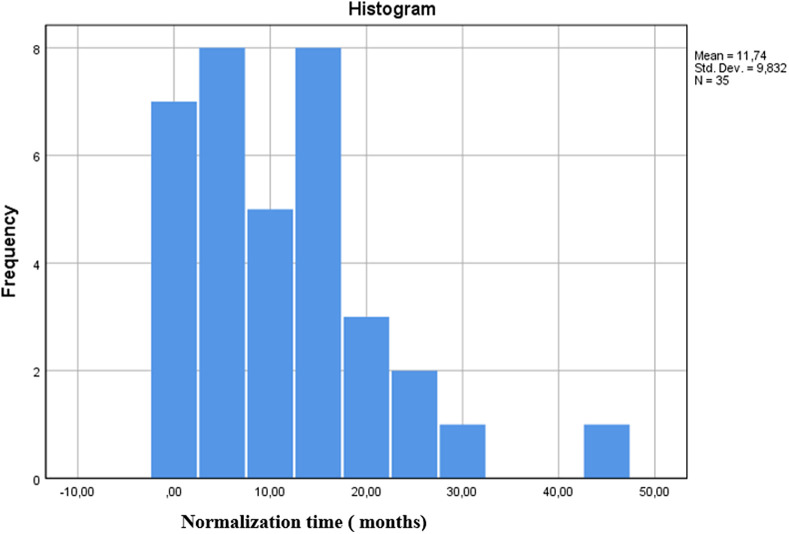
Histogram showing the normalization of serology positivity over time.

When 78 patients in SFPG and CPG were evaluated, the median time to the first positive test was 14 days (0-280). In these 2 groups, only 8 patients were observed to have a single antibody study [2 (2.5%) patients TTG IgA, 6 (7.6%) patients EMA IgA]. Forty-five of the other patients had results for all 4 antibodies studied concurrently in Table 2 Among our patients, the median TTG IgA antibody titer was 200 in CPG with titer rates being 20× ULN. Median TTG IgA antibody titer was 25 in SFPG with titer rates of 2.52× ULN as shown in Table 3. Test normalization in SFPG patients occurs after 20 months during the follow-up period (Figure 1).

Of 25 patients diagnosed with CD, 23 had a concurrent diagnosis of T1DM, and the remaining 2 patients got a CD diagnosis within the first year.

## Discussion

In our study, the rate of EMA and/or TTG antibody positivity was found to be 18.6% of all the patients; 12.6% (53 patients) were serology positive without CD, and 6% were T1DM patients with CD. We found out that the celiac antibodies may be high up to 2.5× ULN at the first presentation of T1DM diagnosis without CD. The majority of the positive antibodies become negative (66%) during the follow-up of 4.1 ± 2.4 years. We realized that it is crucial to initiate the diabetic diet program of the T1DM patients, insulin treatment, follow-up the symptoms, and antibody normalization. The symptoms resolve in a significant proportion of the patients by the time under the diet program with the control of the autoimmune activation of the underlying disease and cytokine storm. We did not recommend an invasive procedure like endoscopic examinations for those patients early at the first presentation of the disease.[Table t3-tjg-34-3-293]

Although CD is common in diabetics, CD serology false positivity is also common among this patient group .^8^ The rate of EMA or TTG antibody positivity in T1DM patients was found to be 9.8% to 20.6% in different studies, and it was found to be 18.6% (78 patients) in our study.^2,9,10^ In the literature, the rate of spontaneous normalization of antibodies reported by Unal et al^11^ was 23.3%, and it was found to be approximately 35.4% in the study by Waisbourd-Zinman et al.^12^

We aimed to determine the characteristics of this SFPG but consistent with the literature, no significant difference was found between CPG and SFPG in terms of clinical and laboratory parameters. Also, the median TTG IgA antibody titer was 200 in CPG with titer rates being 20× ULN. In SFPG, the titer rates are 2.52× ULN. In order to diagnose an asymptomatic CD patient without biopsy from one perspective and in order to recognize SFPG and avoid unnecessary procedures and investigations from the other perspective, we should have some criteria for diagnosis and follow-up in this special patient group.

According to the results of 9 cohort studies by Pham-Short et al.^6^ majority of the patients diagnosed with T1DM concurrently get a CD diagnosis, and the probability of CD development substantially decreases after 5 years. While the previous diabetes diagnosis and treatment guidelines recommended CD screening every 1-2 years, the latest changes updated the frequency of CD screening, so as to be performed at diagnosis, year 2, year 5, and every 5 years thereafter.^8,13^ Studies examining the correlation between the age at diagnosis and the frequency of CD have detected that the less the age at diagnosis is, the higher the CD frequency is.^14^ As the first screening was at the time of diagnosis of T1DM, patients are under a storm of cytokines, intense autoimmune reactions, and glucotoxicity, which may cause erroneous test results. Therefore, we have to differentiate the picture.

A fold rise in the antibody titers may be a clue for the differentiation of SFPG and CD. In their retrospective study in 2020, Travato et al^15^ found the TTG IgA antibody titer being 5-10× ULN and the positive predictive value of EMA IgA positivity to be significant at 0.93. As additional issues such as fasting before anesthesia and difficulty in blood glucose regulation may occur in this patient group during the diagnosis period, we endorse the notion that diagnosis can be made without biopsy in the patient group with serology high positivity as recommended by ESPGHAN 2020 guideline.^5^ A certain titer level should be determined for T1D patients, and serology positivity above that titer should be diagnosed without gastroscopy.

CD screening is performed by endocrinologists in many of the diabetes centers, and it was seen that unnecessary antibody tests are requested in some of them. Although it is not recommended to study TTG IgG and EMA IgG-type antibodies in guidelines, it is noteworthy that TTG IgA and EMA IgA were studied in high percent in our patient group, as it is optional to study these 2 antibodies together in ISPAD guidelines.^8^ As observed in our study, TTG IgG and EMA IgG-type antibody positivity is high in SFPG; therefore, these antibodies should not be used for screening. This notion is supported by the fact that among the patients in 2 groups with celiac antibody positivity in the present study, TTG IgA titer was positive in all of the patients in CPG patients and the presence of IgG antibody positivity, especially in SFPG.

In conclusion, T1DM and CD developing from a common autoimmune basis are concurrently seen as substantially higher compared to the general population, and positivity of CD antibodies studied for screening may be observed early after diagnosis without the presence of the disease.

While the majority of these false positive cases become negative during the follow-up, we should have criteria for this special patient group either to diagnose CD or to recognize false positive serology. This can avoid unnecessary endoscopic procedures in the future.

The present study has some limitations; even if the number of patients with T1DM is high, the number of patients with both T1DM and CD is insufficient to reach a conclusion. Moreover, when assessing the antibody positivity in SFPG, the high rate of TTG IgG and EMA IgG antibody titers being studied caused a drawback. Since diabetic patients are diagnosed and treated first by pediatric endocrinologists, CD screening is also applied by them. Pediatric gastroenterologists evaluate these patients later. For this reason, different screening antibodies were studied by endocrinologists.

## Figures and Tables

**Table 1.** Comparison of the Characteristics of the Patient Groups

	Serology Negative Group (SNG) Median (Min-Max)	Celiac Patient Group (CPG) Median (Min-Max)	Serology False Positive Group (SFPG) Median (Min-Max)	*P*
Age (years)	8.9 (5.6-12.6)	10 (7.2-12.9)	8.8 (6.2-12.00)	.803
HbA1c (%) at diagnosis	12.1 (10.2-13.9)	13.2 (10.9-14.5)	12.7 (10-14.4)	.557
Age	n (%)	n (%)	n (%)	.445
< 10 years	193 (56.9)	12 (48)	28 (52.8)	
≥10	146 (43.1)	13 (52)	25 (47.2)	
Sex (n)	n (%)	n (%)	n (%)	.101
Male	193 (57.1)	13 (52)	22 (41.5)	
Female	145 (42.9)	12 (48)	31 (58.5)	
Hashimoto’s thyroiditis (n)	n (%)	n (%)	n (%)	.071
No	314 (92.6)	23 (92)	45 (84.9)	
Yes	25 (7.4)	2 (8)	8 (15.1)	

**Table 3.** Median Celiac Antibody Titers of the Subjects and Fold Rise in the Antibody Titers

n (25%-75%)	Celiac Patient Group (CPG), (n = 25)	Serology False Positive Group (SFPG), (n = 53)	*P*
Age (years)	10 (7.2-12.9)	8.8 (6.2-12.00)	.559
TTG IgA	200 (151-252)	25.2 (14.4-31.22)	<.001*
EMA IgA	200 (150-320)	33.05 (24.7-48.87)	<.001*
TTG IgA fold rise	20 (15.1-25.2)	2.52 (1.44-3.12)	<.001*
EMA IgA fold rise	10 (7.5-16)	1.65 (1.23-2.44)	<.001*
HbA1c (%) at diagnosis	13.2 (10.9-14.5)	12.7 (10-14.4)	.440

EMA IgA, anti-endomysium IgA; TTG IgA, tissue transglutaminase IA.

*Statistical significance level, *P* <.05 was used for all tests.

**Table 2. t3-tjg-34-3-293:** The Distribution Characteristics of Positivity and Negativity of the Celiac Antibodies in Two Patient Groups With Positive Celiac Antibodies

	Celiac Patient Group (CPG)			Serology False Positive Group (SFPG)		
	Positive	Negative	n	Positive	Negative	n
TTG IgA	20	0	20	11	38	49
TTG IgG	12	3	15	6	36	42
EMA IgA	20	1	21	17	31	48
EMA IgG	11	2	13	12	32	44

EMA IgA, anti-endomysium IgA; EMA IgG, anti-endomysium IgG; TTG IgA, tissue transglutaminase IgA; TTG IgG, tissue transglutaminase IgG.
